# Sequence and structure based deep learning models represent different aspects of protein biochemistry

**DOI:** 10.1101/2023.03.20.533508

**Published:** 2023-03-20

**Authors:** Anastasiya V. Kulikova, Daniel J. Diaz, Tianlong Chen, T. Jeffrey Cole, Andrew D. Ellington, Claus O. Wilke

**Affiliations:** 1 Department of Integrative Biology, University of Texas at Austin, Austin, Texas, USA; 2 Department of Chemistry, The University of Texas at Austin, Austin, TX, USA.; 3 Center for Systems and Synthetic Biology, The Department of Molecular Biosciences, The University of Texas at Austin, Austin, TX, USA; 4 Institute for Foundations of Machine Learning (IFML), The University of Texas at Austin, Austin, TX, USA; 5 Department of Electrical and Computer Engineering, The University of Texas at Austin, Austin, TX, USA

## Abstract

Deep learning models are seeing increased use as methods to predict mutational effects or allowed mutations at various sites in proteins. The models commonly used for these purposes include large language models (LLMs) and 3D Convolutional Neural Networks (CNNs). These two model types have very different architectures and are trained on different representations of proteins. LLMs make use of the transformer architecture and are trained purely on protein sequences whereas 3D CNNs are trained on voxelized representations of local protein structure. While comparable overall prediction accuracies have been reported for both types of models, it is not known to what extent these models make comparable specific predictions and/or generalize protein biochemistry in similar ways. Here, we perform a systematic comparison of two LLMs and one 3D CNN model and show that the different model types have distinct strengths and weaknesses. The overall prediction accuracies are largely uncorrelated between sequence and structure based models. Overall, the 3D CNN model is better at predicting buried aliphatic and hydrophobic residues whereas the LLMs are better at predicting solvent-exposed polar and charged amino acids. A combined model that takes the individual model predictions as input can leverage these individual model strengths and results in significantly improved overall prediction accuracy.

## Introduction

Machine learning models such as neural networks are increasingly being used to computationally explore protein variants suitable for protein engineering and to predict their effects on protein structure and function^1–6^. In fact, several deep neural network models, including large language models (LLMs) and convolutional neural networks (CNNs), have helped with the engineering of proteins with enhanced function/stability^7–10^. Interestingly, the deep learning models employed to date differ substantially in their architectures and, more importantly, the type of input data they are trained on. While LLMs models are typically trained on large amounts of sequence data, CNNs have also been trained on input consisting of protein structures^11^, and models trained on both types of input data have produced meaningful insight on protein variants.

Sequence data is significantly more abundant than structure data, so it would be convenient if models could be trained purely on sequence data. And in fact, sequence-based models, and specifically protein large language models (pLLMs) using transformer architectures, have been successfully employed in a number of contexts, including predicting variant effects and protein fitness^5, 12, 13^, predicting post-translational modifications and biophysical attributes^4^, predicting protein structure^4, 14, 15^, and even predicting entire protein-protein complexes^16^. At the same time, CNNs trained on structure data have been successful in enhancing enzyme function activity by suggesting activity enhancing protein variants^9, 10^. Because sequence and structure-based models learn from different protein representations, it is not clear whether they have inherent differences or instead make substantially equivalent predictions. Additionally, because the different model architectures have typically not been applied head-to-head to the same problem, excellent performance of one architecture in one problem area does not imply that a different architecture might not perform similarly well. Further progress on this question requires a direct comparison of different model architectures on the same problem.

Here, we compare structure and sequence-based models head-to-head on one specific task, the prediction of a masked residue. The structure-based model is a 3D CNN^17^, and we compare it to two sequence-based LLMs, protBERT^4^ and ESM^5^. We find that all these models generally perform the task with similar accuracy. However, because the models differ in how they internally represent protein biochemistry and what they take as input data, they tend to make distinct predictions that reflect different aspects of the underlying protein biochemistry. Further, we show that we can improve model performance by combining the models into a single, joint model. The combined model can compensate for weaknesses of individual models by latching on to different protein features depending on the location of the masked residue within the protein structure.

## Results

**Figure 1.**
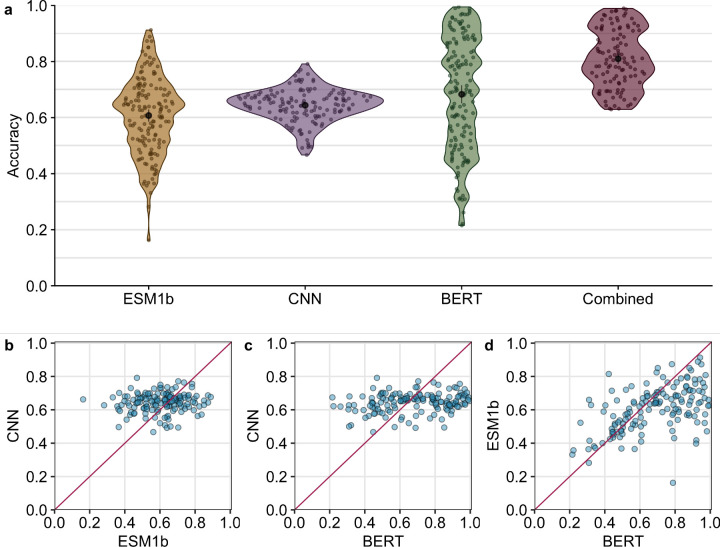
Prediction accuracy compared across models. (a) Average accuracy per protein for each model. The average accuracy is 64.15%, 67.33%, 60.87, and 81.00%, respectively, for the CNN, BERT, ESM1-b, and Combined models. Average accuracies are highlighted by the black point within each violin. (b) Correlation between CNN accuracy and BERT accuracy. (*R*^2^ = 0.0391) (c) Correlation between CNN accuracy and ESM1-b accuracy. (*R*^2^ = 0.0020) (d) Correlation between ESM1b accuracy and BERT accuracy. (*R*^2^ = 0.2645) Each point represents a single protein.

We compared the performance of three self-supervised deep neural network models, two LLMs (ESM1-b^5^ and protBERT^4^) and one 3D CNN^17^, on their training task: predicting masked residues in proteins. Our first question was whether one of the models was consistently outperforming the others. We generated predictions for every residue in a test set of 147 protein structures, chosen such that we minimized overlap with the training data to the extent possible (see Methods for details). We found that the average accuracy across the 147 structures was 60.87%, 64.15%, and 67.33%, respectively, for ESM1b, CNN, and protBERT (Fig. 1a). Here, we defined accuracy as the fraction of correct predictions (top-1) across all residues in a single protein. Importantly, we found that prediction accuracy varied widely across protein structures, in particular for the LLMs, which yielded accuracies as low as 0.2 for some structures and in excess of 0.9 for other structures. Accuracies were more consistent for the CNN model, ranging from approximately 0.5 to 0.8.

We next asked whether the three models had comparable accuracies for the same protein structures. In other words, if one model generated either high or low accuracy for a given protein, did the other models do the same, or did they behave differently? We found that the CNN and the LLMs displayed markedly different behavior. The prediction accuracy of neither of the transformer models was correlated with the accuracy of the CNN model (Fig. 1b, c). By contrast, there was a moderate correlation between the predictions of the two LLMs (Fig. 1d). These findings imply that the two LLMs, which share their input data type and general aspects of their architecture (both are transformer-based language models), display similarities in their predictions but behave by no means identically. And the 3D CNN makes entirely different, uncorrelated predictions.

Because predictions between models were uncorrelated (between transformers on the one hand and the CNN on the other) or weakly correlated (between the two transformer models), we next asked whether we could combine outputs from the three models for improved prediction accuracy overall.

Initially, we tried very simple ensemble methods. One such method was averaging the predictions between the CNN and protBERT model. Specifically, we averaged the predicted probabilities from both models for each amino acid, yielding a final distribution of 20 averaged probabilities. We then took the residue with the highest average probability as the prediction of the combined model and got 19.79% accuracy across the PSICOV proteins. The second method involved taking the highest probability among the two individual models as the final prediction and achieved an average accuracy of 40.19% across proteins. Ultimately, neither method yielded any improvements in overall prediction accuracy. Thus, a more sophisticated ensembling approach was needed.

**Figure 2.**
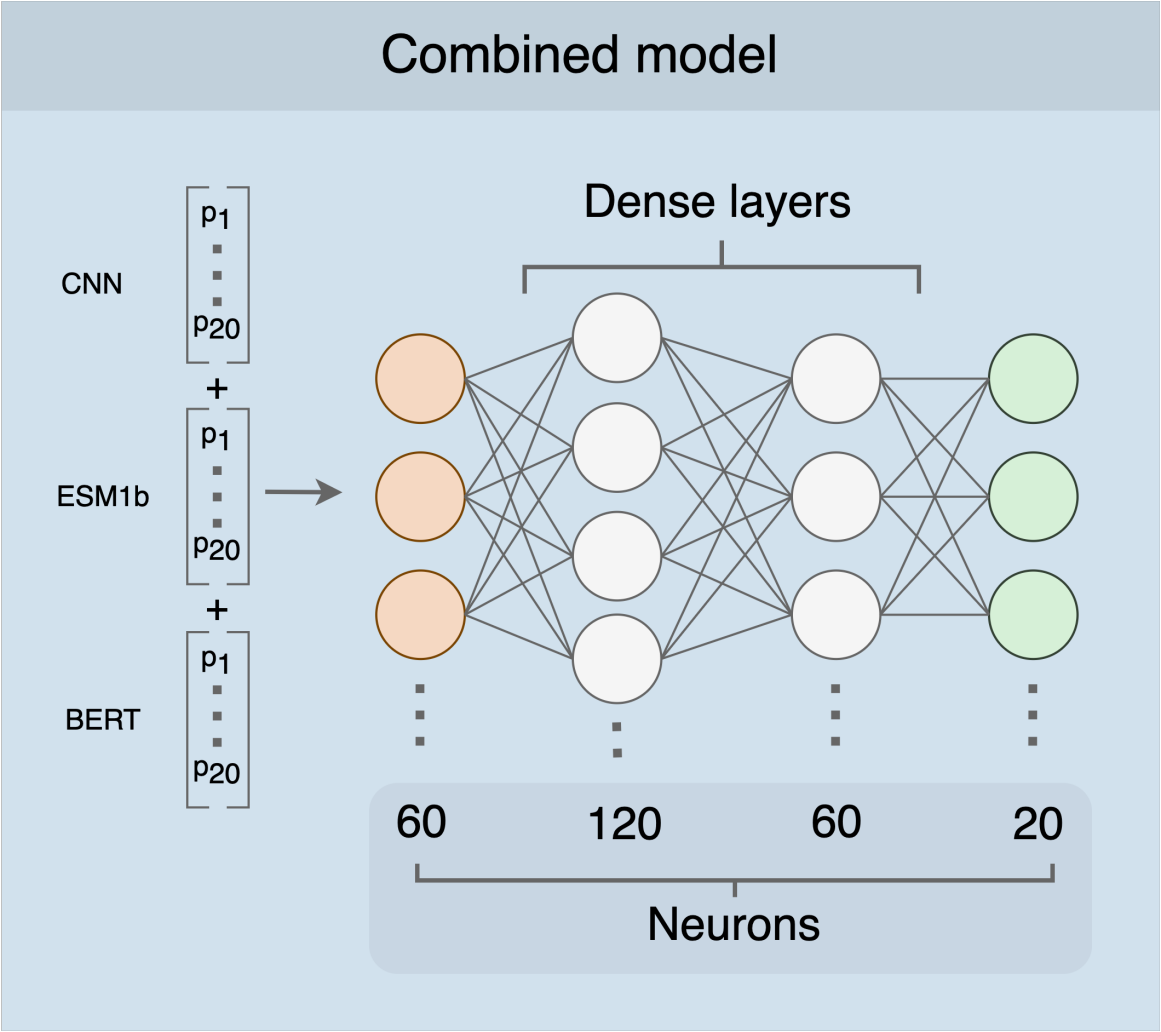
Combined model architecture. As input, the combined model receives a conjoined input vector of length 60 (orange). There are two hidden layers with 120 and 60 nodes, respectively (white). The output is a vector of 20 probabilities, one for each of the amino acids (green).

We next trained a simple fully-connected neural network model to produce a combined prediction from the three individual models. The combined model takes in 60 probabilities (20 per input model), passes them into two intermediate dense layers, and ultimately outputs a set of 20 probabilities, where each node represents the probability of one of 20 amino acids (Fig. 2). We trained this model using a training dataset consisting of 3,209 proteins (Supplementary Figs. S1 and S2). After training, we generated predictions on a test set of 147 proteins and assessed prediction accuracy for each protein. We found that the combined model outperformed all three individual neural networks with an average accuracy of 81%, an improvement of over 10 percentage points from the most accurate individual model (Fig. 1a).

**Figure 3.**
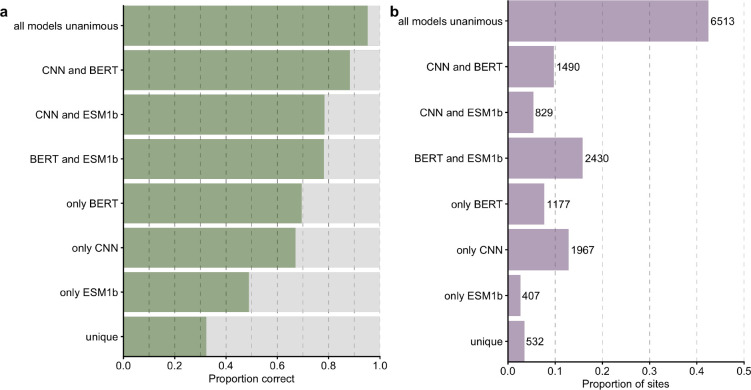
Comparison of combined model predictions with individual model predictions. (a) Proportion of correct predictions for sites at which the combined model prediction agrees with predictions of specific model combinations. (b) Number of sites corresponding to each scenario under part (a).

We then inspected how the accuracy of combined predictions related to the individual predictions. To do so, we classified every individual prediction by whether the combined model agreed with all three individual models, two of the three models, one of the three models, or neither. Unsurprisingly, when all models were unanimous, the prediction accuracy was very high, 95% (Fig. 3a). In other words, whenever the three individual networks individually made the same prediction, and that prediction also coincided with the combined network, then that prediction was likely correct. This scenario was also by far the most common, occurring 42.4% of the time (Fig. 3b). We saw the next highest accuracy whenever the CNN prediction agreed with either one the transformers, closely followed by the case where the two LLMs agreed with each other but not with the CNN (Fig. 3a). For these predictions, the combined model was correct between 80% to 90% of the time. However, agreement between the CNN and just one of the LLMs was relatively rare, and it occurred at fewer than 10% of the sites for each LLM (Fig. 3b). On the other hand, unique predictions (where the combined model did not agree with any of the individual models) were the least likely correct, yet still better than random chance (5%) at an accuracy of ~ 34%.

Thus, all possible combinations of individual model predictions were possible as predictions by the combined model, including the case where the combined model disagreed with all individual models. This observation suggests that the combined model uses a non-trivial strategy to making predictions and does not simply prefer one individual model or apply majority rule.

**Figure 4.**
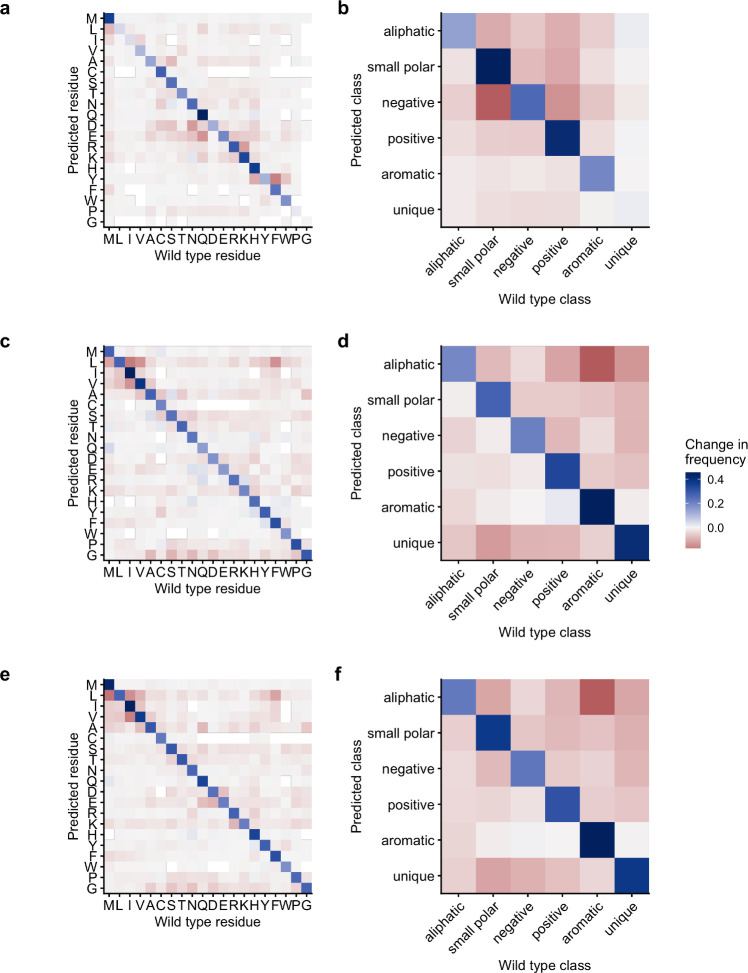
Change in predicted amino acid frequencies from individual models to the combined model. (a) Change in amino acid frequencies from the CNN model to the combined model. (b) Change in amino acid class frequencies from the CNN model to the combined model. (c, d) Change from the BERT model to the combined model. (e, f) Change from the ESM1b model to the combined model.

We next investigated which specific predictions and for which models saw the biggest improvements when going from an individual model to the combined model. We did this by calculating the change in frequency with which we were making correct or incorrect predictions, aggregated either by individual amino acids or by amino acid classes. In all cases, we found correct predictions increased in frequency and mispredictions decreased (blue diagonals and red or white off-diagonals in Fig. 4). However, in general, prediction accuracy increased more when going from one of the LLMs to the combined model than when going from the CNN to the combined model (Fig. 4 c, d, e, f versus a, b). For the LLMs specifically, the combined model dramatically improved predictions for aromatic amino acids as well as unique and small polar amino acids (Fig. 4 c, d, e, f). Going from the CNN model to the combined model, we saw that the greatest improvement occurred for charged amino acids, i.e., for the negative, positive, and small polar amino acids (Fig. 4a, b). These findings suggest that the transformer models perform better at predicting charged amino acids whereas the CNN model performs better at predicting aliphatic core residues.

**Figure 5.**
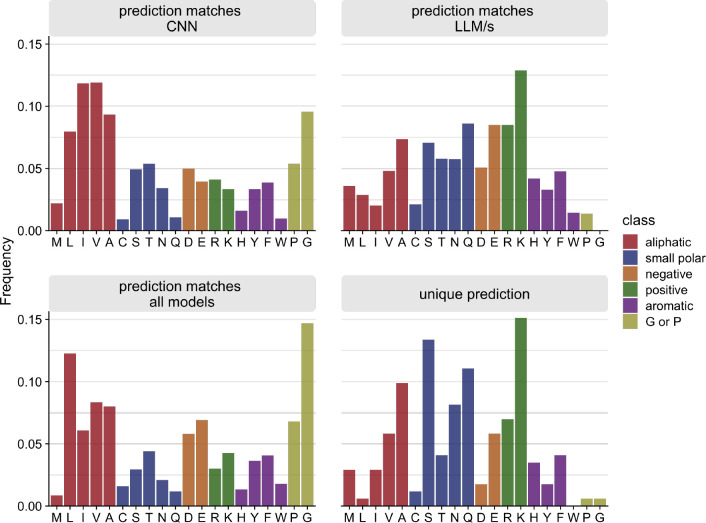
Relative frequencies of correctly predicted amino acids by the combined model, for cases where the prediction of the combined model matches (from left to right and top to bottom) the prediction of only the CNN, only one or both of the LLM models, all individual models, or none of the individual models.

We further confirmed this interpretation of the differences between LLMs and the CNN model by breaking down the distribution of correct predictions by individual amino acids (Fig. 5). This analysis can be thought of as asking which individual model the combined model relied on to make specific predictions. We found that in cases where the combined model relied exclusively on the CNN predictions, the correctly predicted amino acids were more likely aliphatic, whereas in cases where the combined model relied exclusively on predictions from the LLMs, the correctly predicted amino acids tended to be charged (positive or negative) or polar. Somewhat surprisingly, the correct predictions made by the CNN displayed an amino acid distribution most similar to the overall distribution of amino acids within the proteins (Supplementary Fig. S3). The distribution of amino acids when all models were in agreement was somewhat similar (Fig. 5). By contrast, the distribution of amino acids correctly predicted by one or both LLMs were markedly different (Fig. 5). Finally, for correct unique predictions, where the combined model was correct and all individual models where incorrect, the shape of the distribution of amino acids was more similar to the distribution of correct LLM predictions than to the overall distribution of amino acids in the proteins (Fig. 5).

Finally, we asked whether the solvent exposure of a site (i.e., whether it is on the surface or in the core of the protein) contributed to differences in predictions between the models. We did this by correlating the Relative Solvent Accessibility (RSA) of each site in our dataset with the model confidence for that site. Model confidence is defined as the probability of the top-1 prediction at a site, and it correlates well with model accuracy (Supplementary Fig. S4 and prior work^17^). Therefore, we used it here as an approximation of model accuracy, which is not defined for individual sites (a site either is or is not predicted correctly).

**Figure 6.**
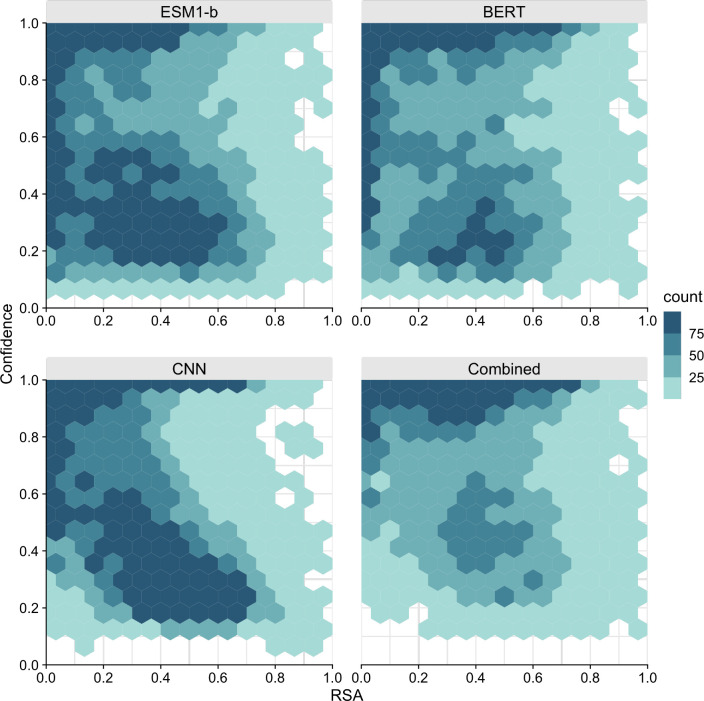
Model confidence as a function of Relative Solvent Accessibility (RSA) for the three individual models and the combined model. Counts are binned into the following four groups: 1–25 predictions/site (lightest color), 26–50 predictions/site, 51–75 prediction/site, and over 75 predictions/site (darkest color).

We found that all models had a tendency to have higher confidence in their predictions for buried residues (RSA near zero) than for exposed residues (RSA of 0.2 or larger) (Fig. 6). However, on average, performance of the LLMs was more uniform across the RSA range (and in particular for the BERT model) than it was for the CNN. The CNN made a large number of low-confidence predictions above RSA of 0.2 but made very few for lower RSA values. In the combined model, we saw prediction confidence markedly increased across all RSA values. The network did not have a strong bias towards either buried or exposed residues and instead made predictions with comparable confidence across the entire spectrum of RSA values.

## Discussion

We have compared the performance of structure- and sequence-based neural network models on the task of predicting masked residues in proteins, and we have found that the different models vary widely in their performance on specific proteins, even if their average accuracies are similar. In particular, predictions by structure-based models are virtually uncorrelated to predictions by sequence-based models. We have further constructed a combined model that uses the predictions of the individual models as input and turns them into an ensemble prediction. This combined model has achieved significantly higher prediction accuracy than the individual models, and importantly, seems to leverage the individual models for their specific strengths. The structure-based 3D CNN performs best at predicting aliphatic core residues, whereas the sequence-based transformer models provide more accurate predictions for polar, solvent-exposed sites. The combined model seems to be able to select the individual model predictions that are most suitable for specific sites. However, on occasion, the combined model also arrives at completely novel predictions that are different from the prediction of either input model.

Sequence-based transformer models and structure-based CNNs differ widely in their training data, their internal structures, and their strengths and weaknesses. The transformer architecture has grown popular in the fields of natural language processing and image recognition^18, 19^. More recently, the self-supervised technique masked-language modeling (MLM) used to train LLMs has been applied to predict masked residues in a protein sequence^3–6^. Such LLMs can capture interactions between amino acids that are far apart in the sequence but spatially close in the structure, relying solely on input sequence for inference, without the need for additional features or annotations^15^. A recent study has shown that a LLMs trained on protein sequences can predict protein structure at the resolution of atoms^15^.

3D CNNs have also been popular in protein research; they have been applied primarily in the context of protein engineering^8–10, 17, 20^. These models are fundamentally different from LLMs as they take the local protein structure rather than the entire amino-acid sequences as input. The input consists of a voxelized box built around a focal residue which is removed before training or inference. This box (or microenvironment) encodes the positions of atoms at the resolution of its individual voxels. CNNs extract the structural features found within these microenvironments, which are then combined and used to predict the focal residue. Thus, only features captured within the given microenvironment participate in residue prediction.

Because LLMs do not have any structural information as input, we might expect them to be consistently worse than 3D CNNs at predicting masked residues. However, we see that this is not the case; both model types can predict residues with comparable accuracies. LLMs compensate for their inherent disadvantage by using much deeper architectures that leverage the attention mechanism, longer training time, and much larger training datasets. As a result, they are able to capture both long- and short-range interactions within a protein^4, 5^, and they are eventually able to learn both sequence-based and structural patterns^15^. On the flip side, structural data directly provides information on physical contacts within a protein, which are most critical for accurate residue prediction^8, 17^. But, structural data provides less information for residues on the surface, which are not strongly constrained by neighboring atoms, and the representation of proteins as independent atoms in space undermines their flexibility as polymers which can shift and rotate to accommodate for new mutations^21^.

The consequence of the differing strengths and weaknesses of the two model architectures is that the different models can be combined for improved overall performance. The network output for all three models consists of a distribution of 20 probabilities, one for each of the amino acids. This distribution of probabilities can also be viewed as an embedding for a biochemical environment^22–24^. Considering that the distributions of probabilities can differ widely even if the same amino acid is predicted to be the most likely, we expect that these distributions carry meaningful information about the microenvironment surrounding the focal residue in the folded protein. Moreover, we would expect similar microenvironments to have similar distributions^25^, which we have previously experimentally demonstrated by transferring mutations predicted on a PETase scaffold to a Cutinase scaffold^9^. Consequently, the combined model can use the probability distributions of the three input models to infer the likely microenvironment of the focal residue and then infer which amino acid to place at this location. This is not a simple majority rule among the models but a non-trivial inference task, as can be seen from the fact that the model on occasion makes unique predictions distinct from any of the input models.

In the combined model, we have noticed that predictions made by the LLMs are used more often for polar/charged amino acids than are the CNN model predictions. This observation can be explained by the fact that the CNN model by itself performs very well on aliphatic and hydrophobic amino acids and poorly on polar and charged amino acids. The CNN, as a structure-based model, is more accurate at predicting core residues, which are surrounded by atoms on all sides, and such residues are primarily non-polar and uncharged. Polar/charged amino acids are often found on the periphery of the protein in areas of high solvent accessibility, where the voxelized input boxes to the CNN are partially empty and thus provide less data to base inference on. The LLMs, on the other hand, do not experience this limitation and are not as biased towards core residues.

One challenge we encountered with LLMs is achieving a clean separation between training and test datasets. Because the LLMs require very large training datasets, they are frequently trained on virtually any unique protein sequence available. This raises the possibility that these models overfit to their training datasets to the point of memorization. With enough training, any model will eventually memorize the training set and be unable to generalize to unseen data^26, 27^. We saw evidence of potential overtraining when we used more recent models of protBERT and esm1-b, as well as esm1-v^6^. For these models, we obtained accuracies averaging at ~95% across proteins (Supplementary Figs. S5 and S6). However, since these models had trained on datasets containing most proteins in the UniRef RCSB databank^28^, we could not guarantee that our test sequences were not in the training data. In fact, we are quite certain they were. Here, primarily for this reason, we used older verisons of protBERT and esm1-b because they were trained on smaller datasets. Ideally, all models would need to be retrained on a clean and consistent dataset for a better comparison.

In summary, our results show that even a relatively simple combined model significantly improves performance relative to the individual models with differing architectures. We have seen how different neural network types, due to differences in how they learn protein features, can make unique contributions to the same residue prediction task. At the same time, predictions that match between a sequence-based and a structure-based model are most likely to be correct, implying that although model predictions are not strongly correlated they do show some overlap. It remains an open question whether LLMs can in principle be trained to the point where they perform as well as or better than a structure-based model, or whether there are inherent differences in these approaches that can never be fully overcome. One conceptual difficulty with this question is that LLMs use such large training sets and so many parameters that they in essence memorize the entire known protein universe. To what extent such models actually generalize protein biochemistry is difficult to assess.

## Methods

We worked with three pre-existing self-supervised deep learning models trained to predict masked residues: two sequence-based transformer models, ESM1-b^5^ and protBERT^4^, and one structure-based 3D CNN model^17^. The two LLMs have been trained on protein sequences, while the 3D CNN model has been trained on protein structures. For all models, we defined model performance as the accuracy with which each model could predict masked amino acids in an array of different proteins.

### Testing Individual Models

We first assessed each model separately, using a test dataset we had previously used in studying the 3D CNN model^17^. Our test set is derived from the PSICOV dataset^29^, which consists of 150 well studied protein structures commonly used for covariation analyses. From this dataset, we removed three proteins for technical reasons, as described^17^. We verified that none of the remaining 147 proteins were in UniRef50, the training set of the esm1-b model. Similarly, the CNN training set had been chosen to not contain proteins with a sequence similarity of greater than 50% to any of the proteins in the test dataset^17^. Because protBERT has been trained on nearly every sequence in the UniRef RCSB databank^28^, we could not guarantee that our test sequences were not in the training data for that model.

LLMs output was generated using the berteome library (version 0.1.6) available at: https://github.com/tijeco/berteome. The berteome library requires prior installation of the transformers package available at: https://pypi.org/project/transformers/. We used version 4.10.0 of the transformers package. The berteome library generates esm1-b predictions from the pre-trained esm1b_t33_650M_UR50S model^5^ and protBERT predictions from the pre-trained Rostlab/prot_bert model^4^. CNN model output was generated using the network with input box size of 20Å^3^ as described^17^.

### Generating a Combined Model

After assessing each individual model, we used the outputs of all three neural networks as training data for a combined model. The combined neural network model consisted of four layers: an input of 60 nodes (20 per model), two intermediate layers of 120 nodes and 60 nodes (“relu” activation function), respectively, and a final output layer of 20 nodes (“softmax” activation function), where each node represents the probability of one of the 20 amino acids. All layers are fully connected. The model was implemented in python (v3.8.9) with the Tensorflow library (v2.9.2) using the keras API (v2.9.0)^30^.

To compile a training set for the combined model we first downloaded all PDB ID’s of proteins clustered into 80% homology groups from the RCSB website using the following link: https://cdn.rcsb.org/resources/sequence/clusters/clusters-by-entity-80.txt. To avoid reusing any proteins previously used to train the individual models, we removed any proteins that were part of the training sets of the 3D CNN (training set as described^17^) and ESM1-b (UniRef50), as well as the test set (PSICOV) and their homologs of 80% or greater similarity. We did not filter out proteins used to train the protBERT model as it was pre-trained on the UniRef100 dataset, containing most of the proteins in the RCSB database. Proteins longer than 1024 residues were also removed due to length restrictions of the ESM1-b model. Finally, we removed protein structures where we could not add hydrogen atoms or partial charges with PDB2PQR (v3.1.0)^31^. The resulting training set contained a total of 3209 protein structures.

To produce training data for the combined model, we generated CNN predictions for each residue in our 3209 protein structure files. Similarly, the sequences corresponding to the 3209 protein structures were used to generate predictions from the protBERT and ESM-1b models. The combined network was trained on each protein/sequence position in this dataset for 70 epochs, on the goal of being able to predict the residue at each position in a protein. We used a fixed learning rate of 0.0001 using the Adam optimizer and the “categorical crossentropy” loss function. Finally, the fully trained combined network was tested against the same set of 147 proteins we had used initially to test individual models.

Relative solvent accessibility values were calculated using freeSASA software^32^ and applying the normalization constants as described^33^.

## Supplementary Material

Supplement 1

## Data Availability

Final data analysis and figure production was performed in R^34^, making extensive use of the tidyverse family of packages^35^. The trained neural network model, analysis scripts, training set, and processed data are available on GitHub: https://github.com/akulikova64/BERT_CNN_comparison/
