# Supplementary material for: Sequence and structure based deep learning models represent different aspects of protein biochemistry
Source: bioRxiv. 2023 Mar 20:2023.03.20.533508. Preprint. [Version 1] doi: 10.1101/2023.03.20.533508 (PMC10055221; doi:10.1101/2023.03.20.533508)
Supplement: Supplementary file 1 [file media-1.pdf]

Supplementary Materials for: Sequence and structure based deep learning models represent different aspects of protein biochemistry

Anastasiya V. Kulikova, Daniel J. Diaz, Tianlong Chen, T. Jeffrey Cole, Andrew D. Ellington, Claus O. Wilke

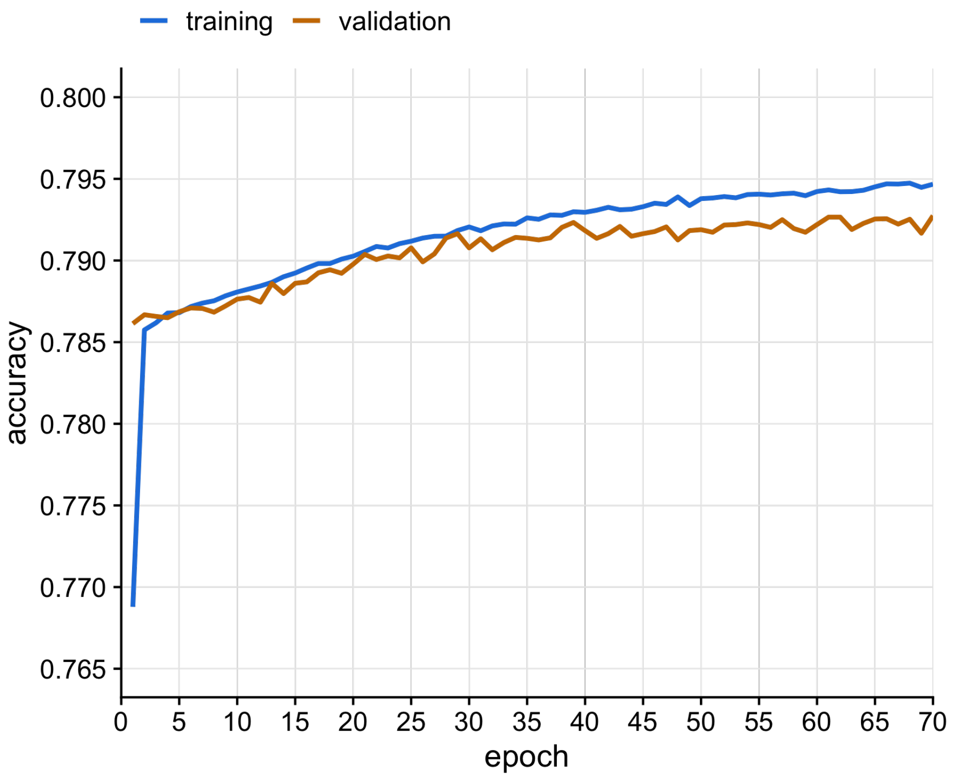

Figure S1. Combined model training and validation accuracy. The plot shows the training accuracy (blue) and the validation accuracy (orange) after each epoch for the combined model starting from epoch 1 to epoch 70. After epoch 1, training and validation accuracy were 76.88% and 78.61%, respectively. After epoch 70, validation accuracy reached 79.27% while training accuracy reached 79.47%.

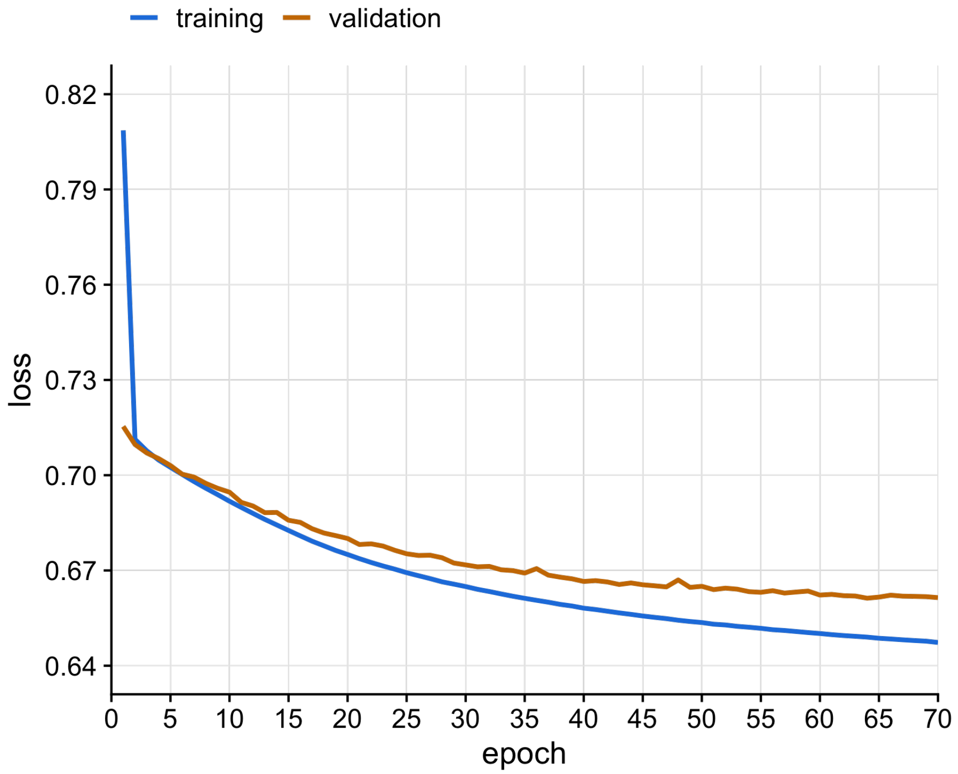

Figure S2. Combined model training and validation loss. The plot shows the training loss (blue) and the validation loss (orange) after each epoch for the combined model starting from epoch 1 to epoch 70. After epoch 1, validation loss was 0.7154, while training loss was 0.8086. After epoch 70, validation loss was 0.6614, while training loss was 0.6473.

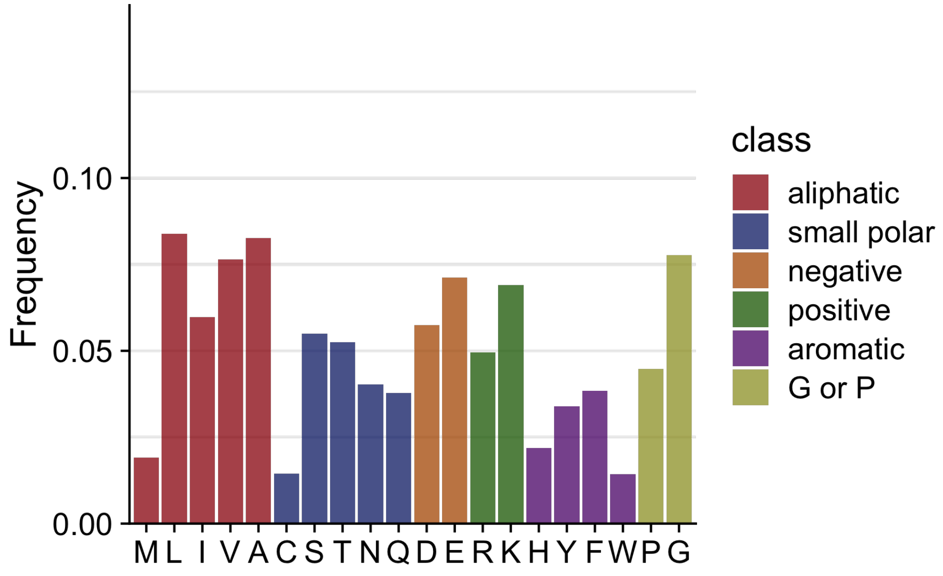

Figure S3. Distribution of wild type amino acids across 147 proteins from the PSICOV dataset.

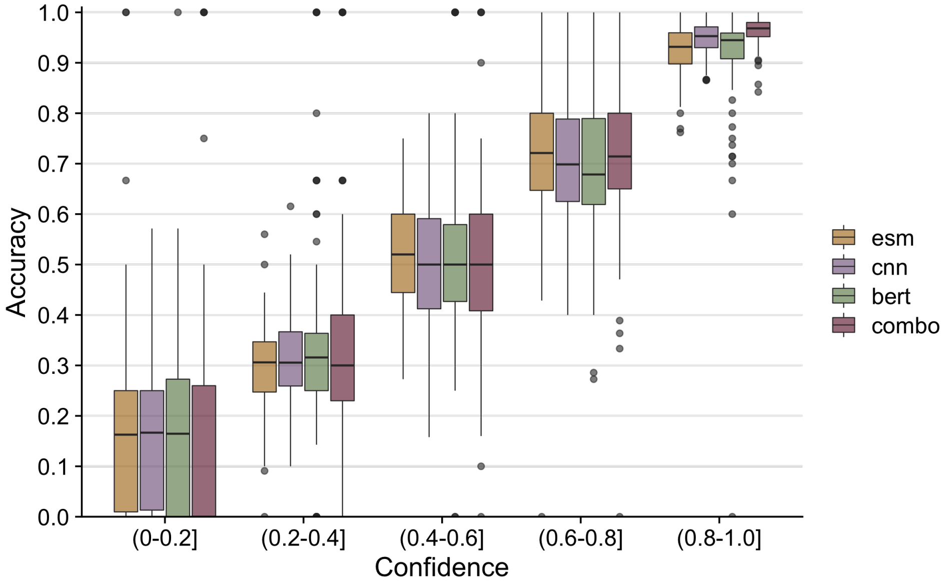

Figure S4. Prediction accuracy as a function of model confidence, for all four models considered in this work.

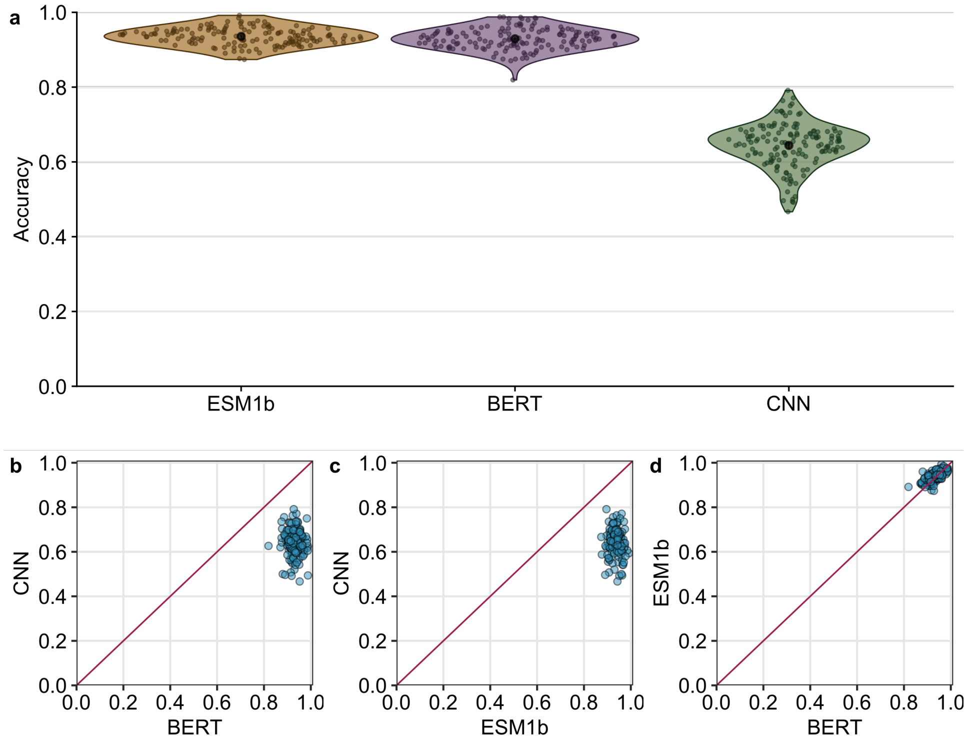

Figure S5. Accuracy of individual models when using newer transformer model versions. (a) Average accuracy per protein for each model. The average accuracy is ∼93% average accuracy for ESM-1b and ∼92% accuracy for the protBERT model and ∼60.7% for the CNN model. Each violin plot contains ∼150 data-points. (b, c, d) Correlations between model types.

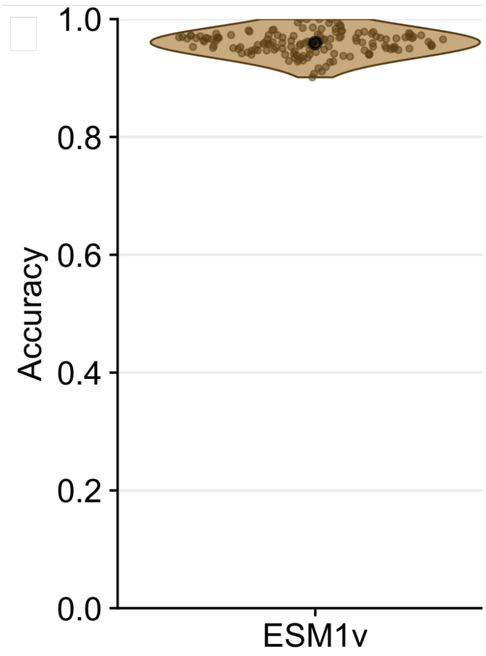

Figure S6. Accuracy of ESM1-v model. When the ESM1-v model (model: esm1v_t33_650M_UR90S_1) was tested on 147 proteins of the PSICOV dataset, the average accuracy for ESM1-v was ∼95%, as indicated by the black point in the center of the violin.

Table S1. Amino acids grouped by class. The following amino acid groups were used in Figures 4 and 5 and are mentioned throughout the paper.

class	included amino acids
aliphatic	Methionine (M), Leucine (L), Inosine (I), Valine (V), Alanine (A)
small polar	Cysteine (C), Serine (S), Threonine (T), Asparagine (N), Glutamine (Q)
negative	Aspartic Acid (D), Glutamic acid (E)
positive	Arginine (R), Lysine (K)
aromatic	Histidine (H), Tyrosine (Y), Phenylalanine (F), Tryptophan (W)
unique	Proline (P), Glycine (G)
